# Hemato-biochemical and pathological changes on avian influenza in naturally infected domestic ducks in Egypt

**DOI:** 10.14202/vetworld.2015.1177-1182

**Published:** 2015-10-09

**Authors:** Essam A. Mahmoud

**Affiliations:** Department of Clinical Pathology, Faculty of Veterinary Medicine, Zagazig University, Zagazig City, Sharkia Province, Egypt

**Keywords:** aminotranseferases, anemia, avian influenza, creatinine, ducks, uric acid

## Abstract

**Aim:**

Few studies have been made in regard to avian influenza (AI) in ducks, thus the aim of this work was planned to investigate the hematological, biochemical, and pathological changes in domestic Egyptian ducks naturally infected with AI.

**Materials and Methods:**

30 duck from private backyards 3-month-old 15 were clinically healthy (Group 1) and the other fifteen (Group 2) were naturally diseased with AI (H5N1). The disease was diagnosed by polymerase chain reaction as H5N1.

**Results:**

Duck showed cyanosis, subcutaneous edema of head and neck with nervous signs (torticollis). Hematological studies revealed a microcytic hypochromic anemia. Biochemical studies revealed a significant decrease in total protein, albumin and globulin concentration with significant increase of activities of aspartate aminotransferase, alanine aminotransferase, alkaline phosphatase, Υ-glutamyl transpeptidase, lactic acid dehydrogenase and creatine phsphokinase. Prominent increase in creatinine and uric acid in addition to hypocalcemia and hyperphosphatemia were significantly detected in the infected ducks. Histopathological finding confirm these investigations.

**Conclusion:**

The highly pathogenic AIV (A/H5N1) became more severe infectious to ducks than before and causes nervous manifestations and blindness which were uncommon in ducks. Besides the significant increases of hepatic enzymes, brain, heart, and renal markers as a response to virus damage to these organs.

## Introduction

Avian influenza viruses (AIV) threaten most of live beings around the world from the onset of the third millennium. This hazard comes from the zoonotic and lethal effect of these viruses. Lately, several zoonotic avian influenza (AI) A strains have been reported to directly infect humans [1]. The spread of highly pathogenic AIV (A/H5N1) from Asia to Africa in 2005 was considered as a global epidemiological twist. The first entrance of A/H5N1 to Egypt recorded in mid-February 2006 which accompanied enormous losses in poultry industry [2]. Highly pathogenic AIV was one of the first viral diseases described in poultry. Infections with AIV of low and high pathogenicity are commonly reported in domestic ducks in many parts of the world [3]. Normally AI viruses, including the highly pathogenic H5 and H7 strains, do not cause disease or death in ducks [4]. Mortality in naturally infected ducks with AI viruses was first recorded during an outbreak of HPAI virus in Italy in 2001 [5]. Since then, a number of H5N1 HPAI viruses cause diseases in ducks [4,6,7]. Clinical signs were recorded on ducks with variable degrees. Ducks appeared more passive, fluffed feathers, conjunctivitis, and slight depression, and progressed over a period to severe neurologic signs consisting of torticollis, in coordination, tremors, cloudy eyes and blindness. The only gross lesion observed at postmortem examination was congested lungs and liver [8].

A highly pathogenic AI (HPAI) A(H5N1) virus was first detected in China in 1996 [9]. The disease (A/H5N1) spread from Asia to Africa in 2005 [2]. The avian influenza (HPAI) viruses cause high morbidity and mortality levels in chickens and ducks and other waterfowl [10,11]. The virus (HPAI) H5N1 transformed from sporadic outbreaks in poultry to persistent circulation in terrestrial and aquatic poultry and potentially in wild waterfowl [12]. In addition leading to, serious economic losses in the poultry industry [13]. Outbreaks associated with the AIV have increased since 2009 [14].

The aim of this study was to investigate the hematological, blood chemistry, and pathological changes in domestic ducks naturally infected with HPAI.

## Materials and Methods

### Ethical approval

Ethical approval was conducted in accordance with the guidelines set by Animals Health Research Ethics Training Initiative, Egypt, and experimental protocols were approved by the Institutional Animal Ethics Committee.

### Animals and methods

30 ducks, 3-month-old, 15 ducks were clinically healthy (Group 1) and the other 15 ducks (Group 2) were naturally diseased with avian influenza (H5N1) were obtained from private backyards in Sharkia governorate and used in this work. Two blood samples were collected at the same time from wing vein from Groups 1 to 2. The first set of blood samples (0.5 ml from each duck) were collected in dipotassium salt of ethylene diamine tetra acetic acid (EDTA) tubes to be used for estimation of the erythrocytic and total leukocytic counts [15], packed cell volume [16] and hemoglobin using the cyanmethemoglobin colorimeteric method [17]. The differential and absolute leukocytic counts were carried out [16]. While the second set of blood samples (5 ml from each duck) were taken without anticoagulant in a sterile test tubes for separation of serum for determination of serum aspartate aminotransferase (AST) and alanine aminotransferase (ALT) [18], *Υ*-glutamyl transpeptidase (GGT) [19], lactic acid dehydrogenase (LDH) [20], creatinine [21], uric acid [22], calcium [23], and inorganic phosphorus [24]. Protein electrophoresis was quantitatively measured [25].

### Histopathological examination

Duck were necropsied and specimens were collected from the liver, kidneys, lungs and trachea. The specimens were fixed in 10% buffered neutral formalin solution, dehydrated in gradual ethanol (70-100%), cleared in xylene, and embedded in paraffin. 5-µ thickness paraffin sections were prepared and then routinely stained with hematoxylin and eosin (H and E) dyes. The sections were mounted with Canada balsam and covered with cover slide to be ready for histopathological examination [26].

### Diagnosis of the disease (polymerase chain reaction [PCR])

Viral RNA were propagated in embryonated chicken eggs for obtaining allantoic fluid samples (HA positive). Allantoic fluid was purified using Gene JET^™^ RNA purification kit (Fermentas) according to the manufacturer’s instructions. The cDNAs were synthesized using Revert Aid^™^ H minus first strand cDNA synthesis kit (Fermentas #K1631) according to the manufacturer’s instructions. Primers used for HA gene amplification are forward H5-kha-1: 5’-CCTCCAGARTATGCMTAYAAAATTGTC-3’ and reverse H5-kha-3: 5’-TACCAACCGTCTACCATKCCYTG-3’ to amplify a portion of the HA gene spanning the HA cleavage site [27]. The PCR was performed in a total volume of 25 µl in a sterile 0.2 ml RNase free PCR tube. The reaction was carried out using thermocycler with the following temperature profile: 3 min at 95°C (initial denaturation) followed by 30 cycles of 30 sec at 95°C (denaturation), 30 sec at 52°C (annealing) and 90 sec at 72°C (elongation), and 15 min at 72°C (final elongation). Amplicons with a size of ~317 bp were visualized by 1.5% agarose gel electrophoresis in Tris-acetate EDTA buffer.

### Statistical analysis

The data obtained from this investigation were statistically analyzed using Student’s t-test [28].

## Results and Discussion

**Figure-1:**
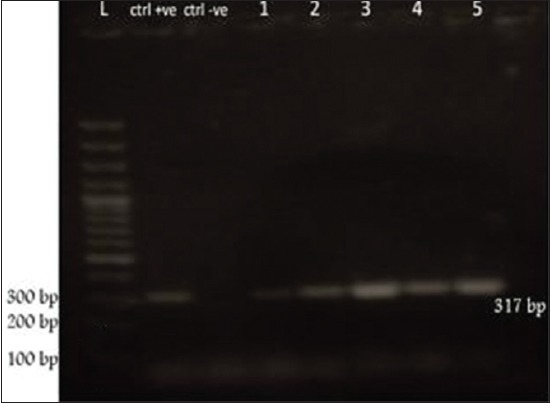
Polymerase chain reaction result.

**Figure-2:**
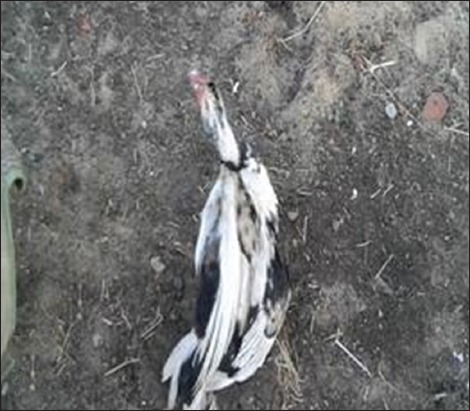
Ducks showed depression and in coordination (Group 2).

**Figure-3:**
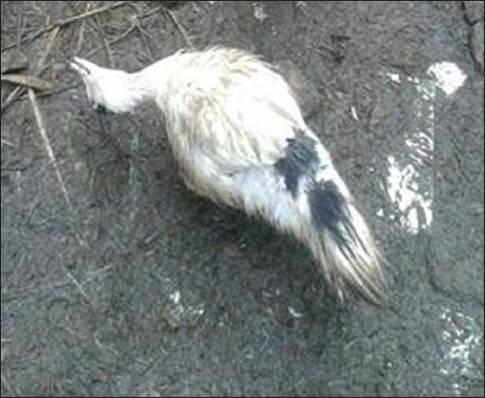
Nervous manifestation (torticollis) (Group 2).

**Figure-4:**
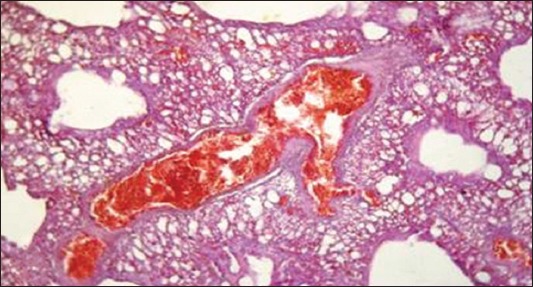
Lung showing severe congestion and hemorrhage (H and E, ×300) (Group 2).

**Figure-5:**
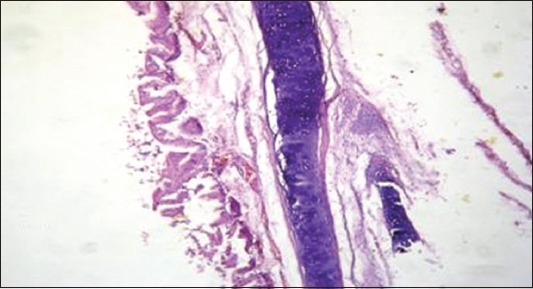
Trachea showing subcutaneous edema, congestion and mild desquamation (H and E, ×300) (Group 2).

Highly pathogenic AIV (HPAIV) H5N1 has been endemic in Egypt since 2006, and there is increasing concern for its potential to become highly transmissible among humans [29]. Ducks were reared in private backyards in Sharkia governorate, Egypt, during December 2014 until February 2015. The recorded clinical symptoms were previously mentioned by several investigators [30,31]. HPAIV H5N1 has been confirmed using PCR (Figure-1). Some ducks developing clinical disease and others remaining sub clinically infected. Such outcome not only depends on the bird species, but also on the infection dose [32]. Clinically, diseased ducks became sick with symptoms including cyanosis, subcutaneous edema of head and neck and nervous signs (torticollis) (Figures-2 and 3) and began to die within 2-3 days of the onset of clinical symptoms. This is confirmed with sever congestion and hemorrhage of the lung and subcutaneous edema [33,34], congestion and mild desquamation of the trachea (Figures-4 and 5). Nervous signs (torticollis) which seen in the diseased duck could be due to the virus reach the CNS through the olfactory nerves, the peripheral nervous system [35], or even via bloodstream [36]. Most recent research [31] concluded that viral antigen were observed in endothelial cells of brain, which provides direct evidence that the HPAIV H5N1 likely invades the CNS by replicating in blood vessels in the brain, which probably one of the main causes for mortality.

Regarding to erythrogram (Table-1) there was a microcytic hypochromic anemia. This anemia could be due to the hemorrhages present in the different organs or due to a decreased erythropoiesis as a result of the kidney damaged. Thrompocytopenia could be due to systemic viral infection [37] which could facilities the hemorrhagic process [38]. While leucopenia (Table-2) is due to lymphopenia and heteropenia which returned to the direct damage effect of the AIV, rather than its replication, depleting the cells from essential components [39]. Also, Acland *et al*. [40] reported a depletion of the lymphocytes from the white pulp of the spleen in avian influenza outbreaks in broiler and layer chickens (H5N3).

**Table-1:** Hemogram of normal and diseased ducks with H5N1 viruses (mean values±SE).

Groups	Parameters					

	RBCs (×10^6^/µl)	PCV (%)	Hb (g/dl)	MCV (Fl)	MCH (pg)	MCHC (%)
1	2.58±0.04	37.75±0.28	12.53±0.04	146.25±2.56	48.57±0.79	33.23±0.24
2	2.15±0.05**	28.90±0.54**	8.45±0.09**	134.86±4.47*	39.47±1.20**	29.39±0.81**
% difference	−16.66	−23.44	−32.56	−7.78	−18.73	−11.55

** Highly significant at p≤0.01, RBC=Red blood corpuscles, MCV=Mean corpuscular volume, Hb=Hemoglobin, MCH=Mean corpuscular hemoglobin, PCV=Packed cell volume, MCHC=Mean corpuscular hemoglobin concentration,

* Significant at p≤0.05

**Table-2:** Leukogram and platelet count of normal and diseased ducks with H5N1 viruses (mean values±SE).

Groups	Parameters (×10^3^//µl)				

	TLC	Heterophil	Lymphocyte	Monocyte	Platelet
1	18.17±0.21	4.75±0.23	11.37±0.30	0.66±0.07	16.10±0.41
2	14.47±0.57**	3.89±0.27*	8.69±0.38**	0.47±0.09^NS^	14.45±0.54*
% difference	−20.36	−18.11	−23.57	−28.79	−10.24

** Highly significant at p≤0.01, TLC=Total leukocytic count, SE=Standard error,

* Significant at p≤0.05

Proteinogram (Table-3) revealed hypoproteinemia, hypoalbuminemia and hypoglobulinemia. The hypoproteinemia and hypoalbuminemia may be due to the decreased feed intake, loss through intestine and/or disturbed metabolism of the liver, in addition to the effect of AIV on the kidneys which leads to albuminuria. Hypoglobulinemia either alpha globulins, beta globulins, or gamma globulins tabulate the immunosuppressive effect of AIV [41].

**Table-3:** Proteinogram of normal and diseased ducks with H5N1 viruses (mean values±SE).

Groups	Parameters (g/dl)					

	Total protein	Albumin	Alpha1 globulin	Alpha2 globulin	Beta globulin	Gamma globulin
1	7.14±0.23	4.41±0.23	0.17±0.02	0.78±0.06	0.79±0.06	0.98±0.04
2	5.45±0.30**	3.50±0.29*	0.12±0.01**	0.53±0.07*	0.48±0.07**	0.82±0.05*
% difference	−23.66	−20.63	−29.41	−32.05	−39.24	−16.32

** Highly significant at p≤0.01, SE=Standard error,

* Significant at p≤0.05

**Figure-6:**
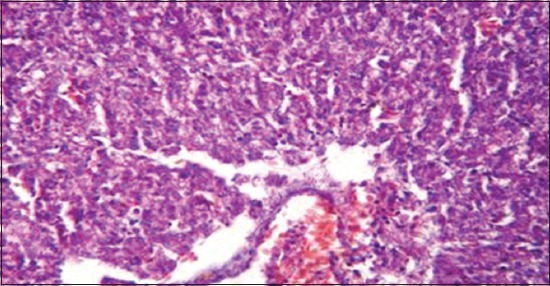
Liver showing degenerative and necrotic changes in hepatic tissue and Hemorrhage in portal area (H and E, ×1200) (Group 2).

**Figure-7:**
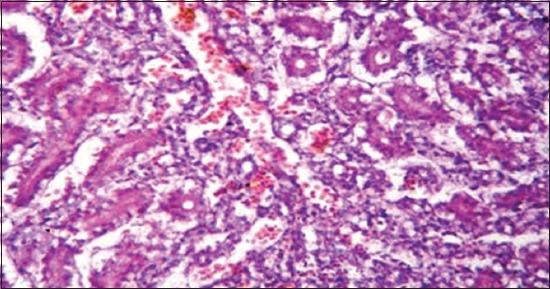
Kidney showing necrotic renal tubule and hemorrhage in renal parenchyma (H and E, ×1200) (Group 2).

Regarding to results of liver function tests (Table-4) revealed a significant increase of aminotransferases activities (AST and ALT) and ALP that could be due to liver involvement [42]. These results were confirmed with the presence of degenerative and necrotic changes in hepatic tissue and hemorrhage in portal area (Figure-6). While a significant increase of GGT, LDH, and creatine phsphokinase may be due to liver, kidney, and/or brain dysfunction [43,44]. On the other side, renal function tests (Table-5) appears a significant increase in both of serum creatinine and uric acid as a result of renal tissue damage [45]. In addition, the presence of hypocalcemia, and hyperphosphatemia in the infected ducks compared with the normal. Hypocalcemia could be returned to decreased calcium absorption from the intestine, increased excretion or may be related to hypoalbuminemia [37,46]. Hyperphosphatemia may be combined to hypocalcemia which in turn led to an increase of the parathormone hormone [37]. These results confirmed by histopathological examination of the kidney which showed necrotic renal tubule and hemorrhage in renal parenchyma (Figure-7).

**Table-4:** Some serum enzymes of normal and diseased ducks with H5N1 viruses (mean values±SE).

Groups	Parameters (U/l)					

	AST	ALT	ALP	GGT	LDH	CPK
1	14.57±0.27	31.88±0.25	130.08±0.48	4.83±0.07	246.73±1.81	267.23±1.37
2	25.62±0.28**	40.09±0.31**	161.49±0.68**	8.12±0.15**	324.41±1.98**	303.61±2.18**
% difference	75.84	25.75	24.15	68.12	31.48	13.61

** Highly significant at p≤0.01, AST=Aspartate aminotransferase, ALT=Alanine aminotransferase, ALP=Alkaline phosphatase, GGT=*Υ*-glutamyl transpeptidase, LDH=Lactate dehydrogenase, CK=Creatine phosphokinase, SE=Standard error

**Table-5:** Some renal function tests of normal and diseased ducks with H5N1 viruses (mean values±SE).

Groups	Parameters (mg/dl)			

	Creatinine	Uric acid	Ca	P
1	0.65±0.02	2.85±0.39	8.51±0.31	4.50±0.12
2	0.79±0.02**	5.97±0.68**	7.65±0.15*	4.99±0.11**
% difference	21.54	109.47	−10.11	10.88

** Highly significant at p≤0.01, P=Phosphorus, Ca=Calcium, SE=Standard error,

* Significant at p≤0.05

## Conclusion

It could be concluded that the highly pathogenic AIV (A/H5N1) became more sever infectious to ducks than before and causes nervous manifestations and blindness which were un common in ducks. Besides the significant increases of hepatic enzymes, brain, heart and renal markers as a response to virus damage to these organs.

## Authors’ Contributions

EAM planned the study design, collected and examined samples, drafted and revised the manuscript, read and approved the final manuscript.
